# BAX activation in mouse retinal ganglion cells occurs in two temporally and mechanistically distinct steps

**DOI:** 10.21203/rs.3.rs-2846437/v1

**Published:** 2023-05-15

**Authors:** Margaret E. Maes, Ryan J. Donahue, Cassandra L. Schlamp, Olivia J. Marola, Richard T. Libby, Robert Nickells

**Affiliations:** Institute of Science and Technology Austria; Harvard University Boston Children's Hospital; University of Wisconsin-Madison; JAX: The Jackson Laboratory; University of Rochester Medical Center; University of Wisconsin Madison

## Abstract

**Background:**

Pro-apoptotic BAX is a central mediator of retinal ganglion cell (RGC) death after optic nerve damage. BAX activation occurs in two stages including translocation of latent BAX to the mitochondrial outer membrane (MOM) and then permeabilization of the MOM to facilitate the release of apoptotic signaling molecules. As a critical component of RGC death, BAX is an attractive target for neuroprotective therapies and an understanding of the kinetics of BAX activation and the mechanisms controlling the two stages of this process in RGCs is potentially valuable in informing the development of a neuroprotective strategy.

**Methods:**

The kinetics of BAX translocation were assessed by both static and live-cell imaging of a GFP-BAX fusion protein introduced into RGCs using AAV2-mediated gene transfer in mice. Activation of BAX was achieved using an acute optic nerve crush (ONC) protocol. Live-cell imaging of GFP-BAX was achieved using explants of mouse retina harvested 7 days after ONC. Kinetics of translocation in RGCs were compared to GFP-BAX translocation in 661W tissue culture cells. Permeabilization of GFP-BAX was assessed by staining with the 6A7 monoclonal antibody, which recognizes a conformational change in this protein after MOM insertion. Assessment of individual kinases associated with both stages of activation was made using small molecule inhibitors injected into the vitreous either independently or in concert with ONC surgery. The contribution of the Dual Leucine Zipper-JUN-N-Terminal Kinase cascade was evaluated using mice with a double conditional knock-out of both *Mkk4* and *Mkk7*.

**Results:**

ONC induces the translocation of GFP-BAX in RGCs at a slower rate and with less intracellular synchronicity than 661W cells, but exhibits less variability among mitochondrial foci within a single cell. GFP-BAX was also found to translocate in all compartments of an RGC including the dendritic arbor and axon. Approximately 6% of translocating RGCs exhibited retrotranslocation of BAX immediately following translocation. Unlike tissue culture cells, which exhibit simultaneous translocation and permeabilization, RGCs exhibited a significant delay between these two stages, similar to detached cells undergoing anoikis. Translocation, with minimal permeabilization could be induced in a subset of RGCs using an inhibitor of Focal Adhesion Kinase (PF573228). Permeabilization after ONC, in a majority of RGCs, could be inhibited with a broad spectrum kinase inhibitor (sunitinib) or a selective inhibitor for p38/MAPK14 (SB203580). Intervention of DLK-JNK axis signaling abrogated GFP-BAX translocation after ONC.

**Conclusions:**

A comparison between BAX activation kinetics in tissue culture cells and in cells of a complex tissue environment shows distinct differences indicating that caution should be used when translating findings from one condition to the other. RGCs exhibit both a delay between translocation and permeabilization and the ability for translocated BAX to be retrotranslocated, suggesting several stages at which intervention of the activation process could be exploited in the design of a therapeutic strategy.

## Background

Proteins of the *BCL2* gene family regulate mitochondrial involvement and dysfunction during the process of intrinsic apoptosis. The family is sub-classified into three distinct groups based on the presence of BCL2 Homology (BH) domains. These include full-length anti-apoptotic proteins (BCL2 and BCL2L1 (BCLX_L_)) and full-length pro-apoptotic proteins (BAX and BAK), which contain all 4 BH domains, and pro-apoptotic activator proteins which contain only the BH3 domain (BH3-only proteins such as BIM, BBC3, BID, BAD, HRK, NOXA, and BMF). Both BAX and BAK are found in an equilibrium between the surface of the mitochondrial outer membrane (MOM) and the cytosol in a process that is controlled by BCL2 and/or BCL-X_L_, which retrotranslocate the pro-apoptotic proteins to the cytosol (1, 2, 3). In this equilibrium, BAX is primarily cytosolic, while BAK is predominantly located on or near the MOM surface. Upon stimulation of the cell death program, one or more BH3-only proteins are either post-translationally modified or expressed. These function to antagonize anti-apoptotic proteins, while also putatively activating pro-apoptotic full-length proteins (4, 5).

The process of BAX activation has been most extensively studied. BAX is translocated to the MOM (6, 7, 8) where it undergoes a conformational change exposing a hydrophobic C-terminal domain allowing it to anchor in the membrane. BAX monomers then dimerize and these begin to oligomerize forming large molecular weight complexes that are often localized to the scission sites of fragmenting mitochondria (9). A principal function of BAX is to permeabilize the MOM (MOMP), which leads to the release of proapoptotic molecules, such as cytochrome c and SMAC/Diablo, that signal caspase activation. Studies in tissue culture cells show that MOMP occurs virtually instantaneously with the initiation of BAX translocation (10, 11), consistent with reports that even a single activated BAX molecule can destabilize a lipid bilayer to create a proteolipid pore large enough to allow passage of cytochrome c (12). An exception to this timing of MOMP is during the process of anoikis, which is apoptosis that is stimulated by loss of cell-cell or cell-matrix adhesion (13). Loss of adhesion induces BAX translocation, but permeabilization is delayed by several hours (14), which allows for BAX retrotranslocation once the cells regain surface contacts.

Retinal ganglion cells (RGCs) are long projection neurons of the central nervous system that transmit light-stimulated electrical signals from the retina to visual centers in the brain through axons that project into the optic nerve. These neurons are highly sensitive to both acute and chronic optic nerve damage, which causes RGC death and blindness in a variety of conditions including glaucoma. Based on prevalence statistics for glaucoma alone, RGC loss represents one of the most common forms of neurodegeneration in humans, projected to affect more than 111 million individuals world-wide by 2040 (15). RGCs die by executing the intrinsic apoptotic program. Evidence for this comes from studies showing that manipulating the balance of BCL2 family proteins provides nearly complete abrogation of RGC soma death in both development and after optic nerve damage.

Overexpression of BCL2 or BCLX_L_ in RGCs yields supernumerary cells in adult mice, indicative of failure of redundant RGCs to undergo developmental apoptosis, and dramatic preservation of RGCs after axotomy and in the DBA/2J mouse model of glaucoma (16, 17, 18, 19, 20, 21). Similarly, genetic ablation of the *Bax* gene yields even greater inhibition of developmental apoptosis (22) and virtually complete elimination of RGC death in mouse models of acute optic nerve crush (ONC) (23, 24, 25) and in DBA/2J glaucoma (26, 27). Importantly, most neurons, including RGCs, are exclusively reliant on BAX because *Bak* transcripts undergo a splice variant that introduces a premature stop codon, eliminating full length BAK as a functioning pro-apoptotic protein (28, 29, 30, 31).

Adult RGCs express select members of the *BCL2* gene family (32). While *Bcl2* is expressed during early development (33), adult RGCs predominantly express B*clX_L_* as the principal anti-apoptotic full length gene (34). Stimulation of RGC death by optic nerve damage is associated with activation of a kinase cascade initiated by Dual Leucine Zipper Kinase (DLK), followed by Mitogen-activated protein Kinase Kinases (MKK4/7), Jun-N-terminal Kinases (JNK2/3) culminating in the activation of the transcription factors p53 and JUN (pJUN) (35, 36, 37, 38, 39, 40, 41, 42). These transcription factors then selectively upregulate the expression of different BH3-only genes (43). Genetic ablation and gene expression studies indicate that RGCs have overlapping or redundant dependence on multiple BH3-only proteins (25, 44, 45, 46, 47, 48).

To date, few studies have examined the process and timing of BAX activation during the progression of RGC death, even though this likely represents the most critical transition point in the commitment of cells to the apoptotic pathway (49). Immunostaining for BAX shows increased staining of RGCs beginning as little as 30 minutes after acute optic nerve damage, peaking at 3–7 days (48, 50). While these results were originally interpreted as showing an increase in BAX expression, they likely reflect the process of translocation and concentration of cytosolic BAX at the MOM of RGCs. Supporting this, more recent studies have shown that forced expression of a GFP-BAX fusion protein in RGCs failed to spontaneously induce BAX translocation and death but confirmed that optic nerve damage induced significant translocation in cells during the 3–7 day interval (25, 32). Longitudinal imaging of GFP-BAX expressing RGCs using blue-light confocal scanning laser ophthalmoscopy also suggested that cells exhibiting translocation remained present for several days before clearance (32).

Since development of neuroprotective therapies for RGCs critically relies on BAX activation, we sought to investigate the kinetics of this process in RGCs using both static and live-cell imaging methodologies. Our results demonstrate that the translocation and permeabilization two steps of BAX activation are mechanistically and temporally distinct in RGCs. While most cells irreversibly translocate, some cells exhibit spontaneous retrotranslocation of BAX in a process that resembles the interruption of anoikis, indicating that translocation and MOMP do not occur simultaneously. Further, we show that BH3-only proteins are critical to drive the translocation step, but are not sufficient to elicit permeabilization, which requires the activation of kinases distinct from the DLK-JNK axis. Together, this work highlights several points of intervention to prevent BAX activation and introduces the possibility of reversing the early stage of translocation.

## Results

### GFP-BAX translocation in RGCs after optic nerve crush

**Figure 1:**
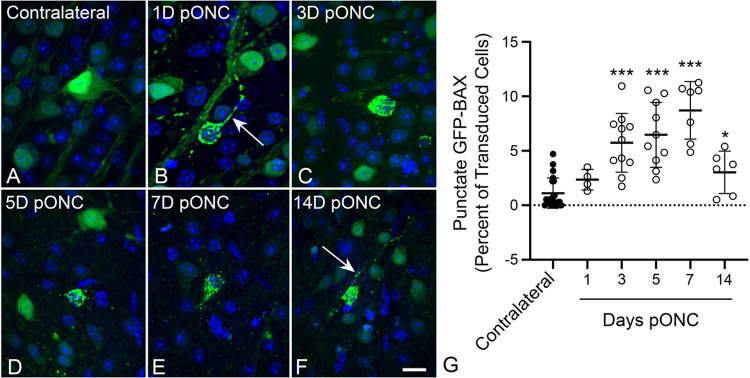
Static imaging of GFP-BAX expressing cells in BALB/cByJ mice showing the time course for GFP-BAX translocation as a function of time after optic nerve crush (ONC). Previously, we have documented that the translocated GFP-BAX forms puncta localized to mitochondria in these cells (25). (A-F) Static imaging of GFP-BAX expressing cells in retinal whole mounts at times post-ONC (pONC) showing localization changes from diffuse to punctate distribution of the fusion protein. In some cells, punctate GFP-BAX is also evident in the axon originating from the cell (arrows in B, F). Scale bar = 20 μm. (G) Graph showing the change in percentage of GFP-BAX transduced cells that exhibit punctate labeling pONC. A significant increase in punctate cells is evident at 3, 5, 7 and 14 days pONC relative to contralateral eyes (*P=0.012, ***P<0.0001, individual *t*-tests). This pattern is consistent with descriptions of BAX accumulation in cells of the ganglion cell layer after optic nerve damage in rats and mice reported by others (25, 32, 48, 50).

RGC susceptibility to ONC is influenced by genetic variation in mouse strains (51, 52). Therefore, we first assessed the time line of GFP-BAX translocation in response to ONC in BALB/cByJ mice, which we planned to use in subsequent live-cell imaging experiments. Adult BALB/cByJ mice were transduced with AAV2/2-*Pgk*-GFP-BAX and one month later were subjected to ONC. Localization of GFP-BAX in RGCs in retinal whole mounts harvested at different times after ONC (Fig. 1A-F) showed an increasing percentage of transduced cells exhibiting punctate localization, indicative of BAX translocation to the MOM (7, 8, 9, 11, 25, 53). A significantly elevated percentage of cells with punctate GFP-BAX, relative to contralateral retinas, was observed by 3 days and persisted through 7 days after ONC before declining (P < 0.0001, *t*-tests, Fig. 1G), similar to other reports in rodents with acute optic nerve damage (32, 48, 50).

**Figure 2:**
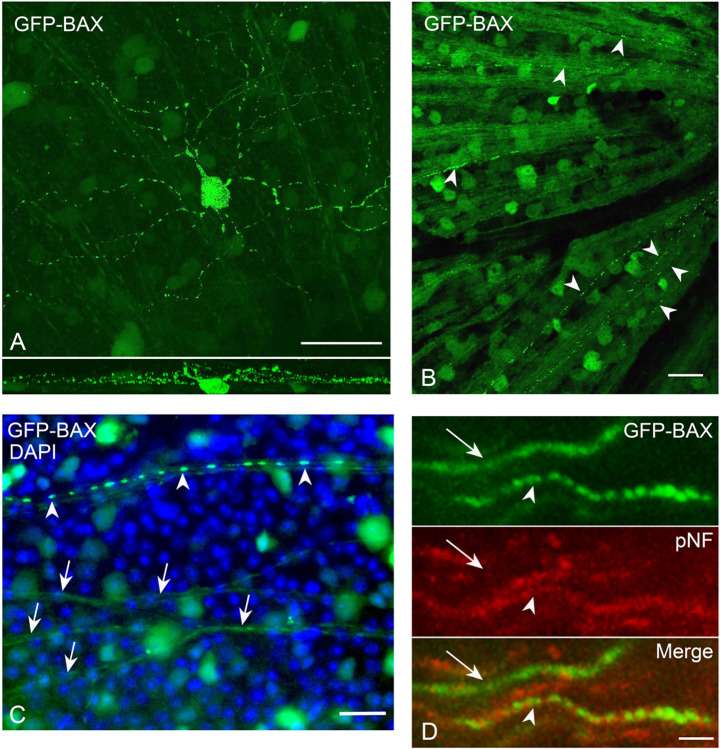
GFP-BAX is translocated in all compartments of retinal ganglion cells (RGCs). (A) A confocal image of a RGC displaying punctate GFP-BAX translocation throughout the dendritic arbor and soma (the intraretinal axon was not conclusively identified in this image) after optic nerve crush (ONC). The lower panel shows the Z-plane of the stack. Given the size and arbor stratification, this likely represents an a-ON sustained RGC. Scale Bar = 50 μm. (B) Retinal whole mount showing intraretinal axons filled with GFP-BAX near the optic nerve head (right). Several axons exhibit punctate localization of GFP-BAX (arrowheads). Scale bar = 20 μm. (C) Higher magnification of a whole mounted retina comparing diffusely labeled axons (arrows) and a punctate labeled axon (arrowheads). Punctate labeled intraretinal axons could be identified in retinas at all time points after ONC examined. Scale bar = 25 μm. (D) Punctate labeling of GFP-BAX in axons of the optic nerve distal to the optic nerve head. Cell death in this experiment was induced by intravitreal injection of staurosporine 24 hrs previously in order to preserve axon integrity with the RGC soma. An axon with diffuse GFP-BAX (arrow) is compared to an axon with punctate GFP-BAX (arrowhead). Note that the axon with punctate GFP-BAX also counterstains with an antibody against phosphorylated neurofilament (pNF) consistent with reports that degenerating neurons, including RGCs, accumulate this antigen (54, 126). Scale bar = 4 μm.

Examination of punctate GFP-BAX localization also revealed that many cells exhibited this feature not only in the soma, but in different RGC compartments, including the dendritic arbor and the intraretinal axons proximal to the crush lesion (Fig. 1B, Fig. 2A-C). To determine if BAX could also be translocated in axons distal to the glial lamina of RGCs undergoing apoptosis, we initiated apoptosis at the level of the soma by intravitreal injection of staurosporine (STS). Sections of the optic nerve of GFP-BAX expressing mouse eyes showed axons with patterns of diffuse BAX and axons with BAX puncta, the latter of which co-localized with the accumulation of phosphorylated neurofilament commonly exhibited by degenerating RGC axons (54) (Fig. 2D). These results indicate that multiple RGC compartments can exhibit BAX translocation in response to an apoptotic stimulus.

#### Live ex vivo imaging of RGCs reveals reduced GFP-BAX translocation kinetics

**Figure 3:**
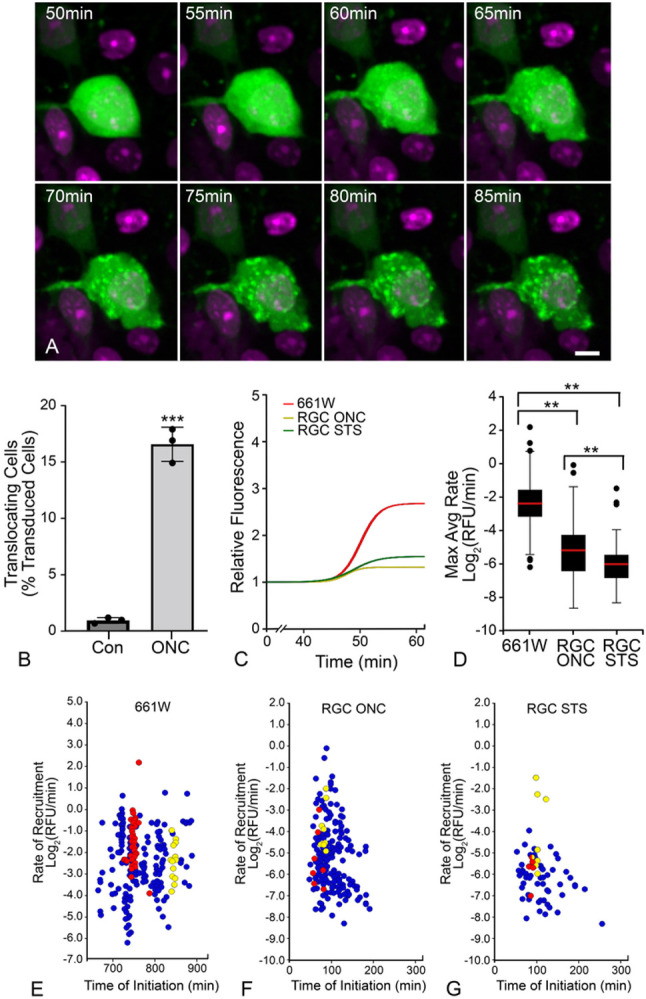
*Ex vivo* live cell imaging of GFP-BAX translocation in retinal ganglion cells (RGCs) of retinal explants. (A) Still images of a GFP-BAX expressing RGC going punctate during a live-cell imaging session (see supplemental video S1). The time stamp refers to the elapsed time from the initiation of the imaging session. DRAQ5 counterstaining was used to highlight nuclei. Scale bar = 5 μm. (B) Graph showing the conversion of imaged cells going from diffuse to punctate in retinas from optic nerve crush eyes (ONC) relative to contralateral eyes (Con). Significantly more cells converted to punctate GFP-BAX in the ONC retinas compared to contralateral retinas during the imaging period (***P>0.001, *t*-test), indicating that initiation of GFP-BAX translocation was primarily reliant on previous ONC. (C) Graph showing the median curves of GFP-BAX translocation comparing RGCs after ONC, naïve RGCs exposed to 1 μM staurosporine (STS) during the imaging session and differentiated retinal precursor tissue culture cells (661W) induced for apoptosis by the expression of histone deacetylase 3 (HDAC3) (31). The time scale (X-axis) has been normalized to show a common time for initiation of BAX accumulation. Raw accumulation curves are shown in supplemental Figure S1. (D) Box and Whisker (5% and 95% limits shown in bars) plots of the maximum rates of GFP-BAX accumulation for all groups shown in (C). The rate of GFP-BAX accumulation in RGC somas is slowest after STS exposure (**P<0.001, *t*-test), but both STS and ONC induce a slower rate of accumulation than that observed in 661W cells (**P<0.001, individual *t*-tests). Of note, 661W cells exhibit a rate of BAX accumulation that is consistent with multiple different lines of tissue culture cells undergoing apoptosis (Table 1). (E-G) Scatter plots showing the max rate versus time of initiation for individual BAX puncta in the 3 groups. The time scale for graph (E) indicates the time after transfection of differentiated cells with a plasmid expressing human HDAC3. The time scale for graphs (F and G) was set to “0” when imaging of the explant began. Foci from 2 individual cells are plotted as red and yellow circles, respectively. The more stacked the alignment of points relative to the X-axis is indicative of simultaneous initiation of GFP-BAX translocation at all sites within a cell. The broad range of points relative to the Y-axis is indicative of variable rates of translocation at different mitochondrial foci within a single cell. Analysis of the coefficient of variation for both initiation and rate indicate that RGCs exhibit greater variability in initiation times and less variability of the intracellular rates, than 661W cells (see Supplemental Figure S2).

To measure the kinetics of GFP-BAX translocation in RGCs, we transduced RGCs in BALB/cByJ mouse retinas using AAV2/2-*Pgk*-GFP-BAX and performed ONC one month later. After 7 days, retinas were harvested and immediately prepared for *ex vivo* live-cell imaging. GFP-BAX translocation occurred in the RGCs of the crushed retina as visualized by the transition from a diffuse to a punctate localization (Fig. 3A, Supplemental Video S1). Contralateral retinas showed no significant increase in the number of imaged cells exhibiting punctate GFP-BAX localization during an imaging session of 3 hours (Fig. 3B). In contrast, a significant proportion of cells in the ONC retinas converted to punctate GFP-BAX localization (P < 0.0001, *t*-test, Fig. 3B), indicating that translocation *ex vivo* was a consequence of ONC *in vivo* and not the process of preparing the retinas for imaging. To provide comparison for GFP-BAX translocation kinetics in ONC-induced mouse RGCs, we also conducted kinetic analyses in mouse RGCs from retinas exposed to the apoptosis-inducer STS, or in differentiated 661W cells overexpressing histone deacetylase 3 (HDAC3). HDAC3 is selectively toxic to differentiated 661W cells (31) and is known to play an important role in RGC pathology after ONC in mice (55, 56, 57, 58).

Previous analyses of BAX translocation kinetics in 3 different tissue culture cell lines showed average rapid rates of BAX translocation (between −1.0 and −1.8 Log_2_ relative fluorescence units (RFU)/min), that was initiated nearly simultaneously at different mitochondrial loci within a single cell (Initiation coefficient of variation (CoV) values between 1% and 5%). Within any given cell, however, the rates of translocation at individual loci exhibited a high degree of variation (Rate CoV values between 47% and 150%) (Table 1). Differentiated 661W cells exhibited a comparable mean rate of translocation (−2.2 Log_2_RFU/min) compared to other *in vitro* cell-experiments (Table 1), along with a low CoV (1%) for initiation times within a given cell and a high CoV (53%) for intracellular rates (Table 1). In contrast, analysis of GFP-BAX translocation in *ex vivo* RGCs yielded several differences compared to *in vitro* tissue culture cells. Regardless of the damaging stimulus (ONC or STS injection), RGCs yielded less overall translocation of GFP-BAX to the MOM than 661W cells (RFU Y-Maximum, Table 1, Fig. 3C, Supplemental Figure S1), which may reflect differences between imaging the RGCs in a thick segment of retina versus tissue culture cells adherent to a coverslip. RGCs also exhibited significantly slower rates of translocation (−5.1 and −6.0 Log_2_RFU/min for ONC and STS respectively, Table 1, Fig. 3D, P > 0.001, individual *t*-tests) than the translocation rates for differentiated 661W cells. Intracellular kinetics were also different for RGCs. The initiation CoV (12–13%) was significantly greater than for 661W cells (P < 0.0001, ANOVA) while the rate CoV (20 – 13%) was significantly smaller (P < 0.0001, ANOVA, Figs. 3E-G, Table 1, Supplemental Figure S2). These data demonstrate that the kinetics of intracellular initiation and rate of translocation, both as a global average and as independent events within a single cell, do not directly translate between cells *in vitro* and cells contained in a complex tissue.

### A subset of ONC-induced RGCs exhibit retrotranslocation of translocated GFP-BAX

**Figure 4:**
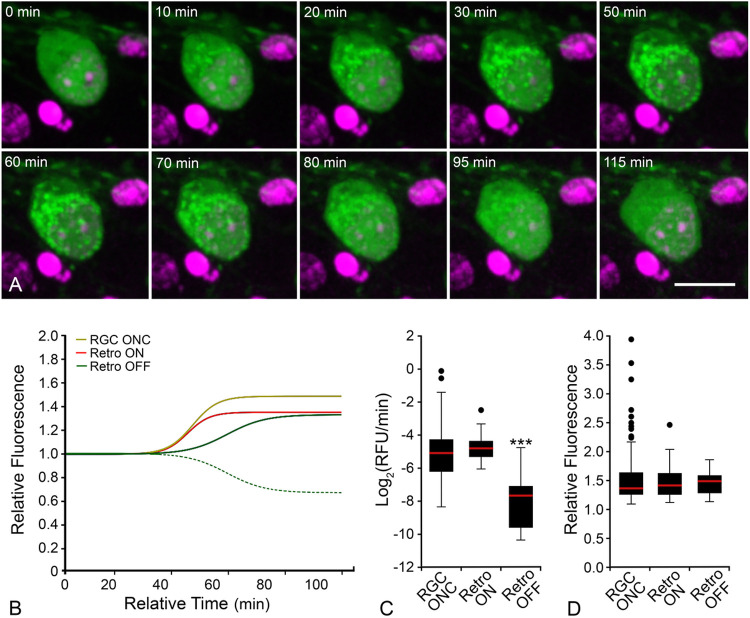
*Ex vivo* imaging shows some RGCs exhibit translocation followed by retrotranslocation. (A) Still images from a RGC that exhibited retrotranslocation during a live-cell imaging session (see supplementary video S2). The time stamp refers to the elapsed time from the initiation of the imaging session. DRAQ5 counterstaining was used to highlight nuclei. Scale bar = 10 μm. (B) Median curves of all data showing that “ON” rates (maximum linear slope) are equal between RGCs with stable translocation and retrotranslocating RGCs. The hashed green line shows the actual median curve of the OFF retrotranslocation data, while the solid green line is the same data that has been flipped on the vertical axis to better highlight the maximum rate compared to the ON rates. The “OFF” rate is slower implying a distinct mechanism driving the retrotranslocation. (C) Box and whisker plot of all rates. The “ON” rates of GFP-BAX translocation is the same comparing stable RGCs and RGCs that exhibit retrotanslocation (P=0.21). The “OFF” rate of GFP-BAX in these cells, however, is significantly slower than both “ON” rates (***P<0.0001, ANOVA). (D) Box and whisker plot of final relative fluorescence showing that stable cells and retrotranslocating cells accumulate similar levels of BAX, while the amount of BAX retrotranslocated is equal to the amount of BAX that was originally translocated in these cells.

Live imaging of GFP-BAX translocation in AAV2/2-*Pgk*-GFP-BAX transduced retinas showed that some RGCs (5/33 cells) in ONC-induced retinas exhibited an unexpected phenomenon. GFP-BAX translocation was followed almost immediately by its retrotranslocation back into the cytosol (Fig. 4A, Supplemental Video S2). Kinetic analysis of the GFP-BAX translocation (ON) rate of these cells was statistically similar to RGCs that translocated GFP-BAX and stably maintained a punctate phenotype. However, the rate of retrotranslocation (OFF rate) from the same cells was significantly slower than the ON rates for both stably converted or retrotranslocating RGCs (P < 0.0001, ANOVA, Figs. 4B and C, Supplemental Figure S3, Table 1). Measurements of the difference in total amount of GFP-BAX fluorescence indicated that retrotranslocating cells translocated a similar amount of GFP-BAX as stably converted cells, and that the process of retrotranslocation removed translocated GFP-BAX to a level indistinguishable from cytosolic localization (Fig. 4D, Table 1).

### RGCs with translocated BAX exhibit a delay before activation of MOMP

**Figure 5:**
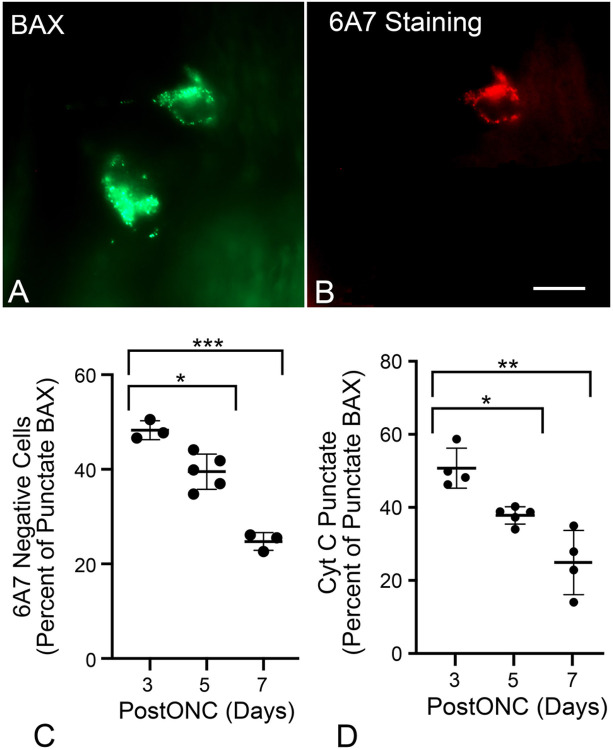
Translocated BAX exhibits a delay before being activated. (A,B) Image of 2 cells exhibiting punctate localization of GFP-BAX induced by ONC and counterstained with the 6A7 monoclonal antibody that recognizes an epitope of BAX that is exposed after activation. Only 1 of the 2 cells is clearly labeled by the antibody. Scale bar = 15 μm. (C) Quantification of the percentage of punctate GFP-BAX cells that are negative for 6A7-staining at times after optic nerve crush (postONC). The mean percentages of cells that are paused for GFP-BAX activation are 48.27% at 3 days after ONC, which decreases to 39.51% at 5 days and 24.73% at 7 days. (D) Scatter plot showing the percentage of cells with punctate mCherry-BAX that also have punctate cytochrome c (Cyt C)-GFP (see Supplemental Figure S3) at days 3, 5, and 7 after ONC. The percentage of paused cells is 50.91% at 3 days after ONC and drops to 38.01% and 25.16% on days 5 and 7, respectively. (*P<0.001, **P=0.0025, ***P<0.0001).

Studies of BAX activation in tissue culture cells indicate that BAX-mediated MOMP events such as cytochrome c release (10, 11, 59, 60, 61) occur rapidly at the onset of BAX translocation to the MOM. The conformational change in BAX that facilitates MOM insertion also exposes its N-terminal domain, which can be detected with the 6A7 monoclonal antibody (8, 62, 63, 64) (Fig. 5A, B). When we assessed 6A7 immunostaining in GFP-BAX expressing RGCs after ONC, approximately 50% of cells with punctate GFP-BAX were 6A7-negative at 3 days post ONC, which progressively decreased over 5 and 7 days (Fig. 5C). Importantly, while we did observe some 6A7-positive cells that were negative for the GFP-BAX fusion protein, we did not observe any 6A7-positive cells that exhibited diffuse localization of the fusion protein. These results suggest that some RGCs exhibit a delay in the permeabilization step of BAX activation, which would result in a delay of MOMP and the release of cytochrome c. To confirm this, we injected mouse eyes with AAV2/2-*Pgk*-mCherry-BAX and AAV2/2-*Pgk*-cytochrome c-GFP, which resulted in co-transduction in 80.9 ± 5.1% of cells (n = 9 retinas, data not shown). Retinal whole mounts harvested at 3, 5, and 7 days after ONC were assessed for cytochrome c-GFP release in cells with punctate mCherry-BAX localization. As with the scoring of cells for 6A7 immunostaining, we observed the same inverse correlation between time and the proportion of cells with punctate BAX that exhibited mitochondrial localization of cytochrome c (Fig. 5D, Supplemental Figure S4).

### BAX translocation and permeabilization in RGCs is influenced by signaling pathways associated with anoikis

**Figure 6:**
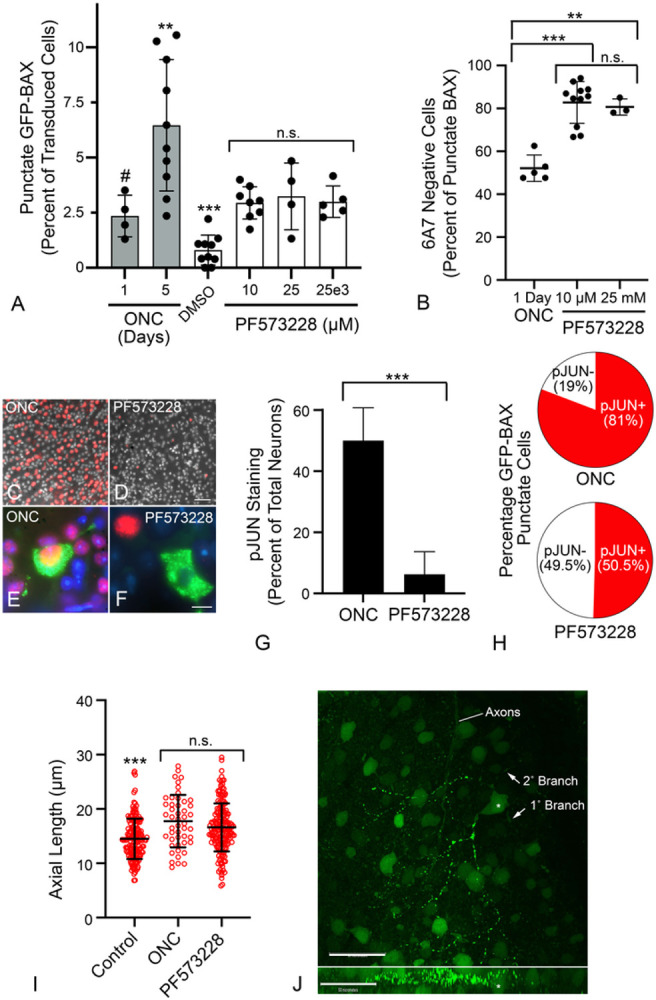
Characteristics of BAX translocation and activation in cells after direct inhibition of Focal Adhesion Kinase (FAK) with PF573228. (A) Quantification of the percentage of GFP-BAX cells that translocate BAX 1 day after intravitreal injection of PF573228. Data are compared to percentages of cells induced by optic nerve crush (ONC) shown in Figure 1 (grey bars). PF573228 induces an increase in the formation of punctate cells that is significantly greater than DMSO vehicle injections alone (***P<0.001), but the level is irrespective of the dose of PF573228 injected (n.s., not significant). PF573228 induces a similar level of translocation observed 1 day after ONC (#P=0.6404, ANOVA), but less than observed 5 days after ONC (**P=0.0031). (B) Translocated BAX induced by PF573228 is predominantly in the paused state (6A7 staining negative) 1 day after injection, at levels significantly exceeding those observed 1 day after ONC (**P=0.0004, ***P<0.0001, individual *t*-tests). (C,D) Retinal whole mounts 1 day after ONC (C) or PF573228 injection (D) stained for pJUN (red). Both treatments induce pJUN accumulation in cells of the ganglion cell layer, although they are more abundant in ONC. Scale bar = 60 μm. DAPI counterstain. (E, F) High magnification of two RGCs exhibiting punctate GFP-BAX (green) and exhibiting robust pJUN accumulation (E) or absence of pJUN accumulation (F). Scale bar = 7 μm. DAPI counterstain. (G) Histogram showing the mean percentage of total neurons expressing pJUN 1 d after ONC compared to 1 d after PF573228 injection (mean ± SD, n=3 and 4 retinas, respectively). (***P<0.0001, *t*-test). (H) Pie charts showing the distribution of cells with punctate GFP-BAX that exhibit pJUN accumulation (red sectors) in both ONC and PF573228 retinas (n=3 and 4 retinas, respectively). Significantly more cells in ONC retinas with BAX translocation were also pJUN positive (P<0.0001, c^2^ test). (I) Scatter plots of maximal axial length of GFP-BAX transduced cells. Both ONC and PF573228 induce BAX translocation in larger cells relative to the population of transduced cells with diffuse BAX in control retinas, 1 day after ONC or injection (ANOVA, ***P<0.0003). There was no significant difference (n.s., *t*-test, P=0.119) between cell sizes in the two experimental groups. Cohorts contained between 48-361 cells measured from retinas of minimum of 3 mice per group. (J) Confocal Z-stack of a cell with partial GFP-BAX translocation in its dendritic arbor induced by PF573228 injection (Z plane shown in lower panel). An asterisk indicates the cell soma. Translocation is observed in regions of the arbor but is not fully extended to the soma, other primary branches and some secondary branches from a branch that is punctate. Axons from different labeled RGCs are indicated. Scale bars = 50 μm.

The delay between BAX translocation and permeabilization, and the phenomenon of retrotranslocation, are features associated with anoikis (13). In tissue culture cells, disruption of cell contacts results in a loss of signaling by Focal Adhesion Kinase (FAK), stimulating BAX translocation through the activation of latent BMF, a BH3-only protein (65, 66). To assess whether BAX activation in RGCs can be driven by anoikis, we first determined if FAK inhibition could induce GFP-BAX translocation and permeabilization in naïve retinas. Figure 6A shows the induction of GFP-BAX translocation in a small percentage of RGCs in response to increasing concentrations of the FAK inhibitor PF573228. Maximum translocation was achieved 24 hrs after injection of the lowest dose of inhibitor (1 μL of a 10 μM solution injected). This percentage was comparable to the percentage of translocated cells in retinas 24 hrs after ONC, but less than the level observed by 5 days after ONC. No significant change in the percentage of translocating cells was observed up to 5 days from the single injection (data not shown) suggesting either clearance of the inhibitor, or that PF573228 was affecting a subset of RGCs. Examination of translocated cells in 10 μM PF573228-injected eyes showed that an average of 82.79% of RGCs with translocated GFP-BAX were 6A7 negative, while only 50% of ONC-induced RGCs were 6A7-negative (P < 0.0001 for 10 μM injection, *t*-test, Fig. 6B). These results suggest that FAK inhibition may primarily induce translocation but not MOM permeabilization.

To address whether FAK inhibition induces known signaling pathways linked to RGC death, we examined the DLK-JNK canonical signaling pathway, which has been widely linked to apoptosis in RGCs after optic nerve damage (35, 37, 38, 39, 40, 41, 67, 68). Importantly, this axis contributes to the up-regulation of several BH3-only proteins that participate in RGC death through the activation of both JUN- and p53-mediated transcription (41, 42, 43, 69, 70, 71). Analysis of transcript abundance for pJUN- and p53-regulated genes showed no significant accumulation of mRNAs from these target genes after PF573228 injection, compared to significant induction in retinas one day after ONC (Supplemental Figure S5). Immunolabeling for accumulating pJUN, however, did show that PF573228 induced the accumulation of this activated transcription factor within 24 hrs after injection (Fig. 6C-F) in a fraction of cells (6.27% of total neurons) albeit in significantly fewer cells than ONC after the same time period (Fig. 6G) (P < 0.0001, *t*-test). Aligning with both reduced pJUN immunostaining and pJUN-mediated gene expression observations, PF573228 induced translocation of GFP-BAX in significantly more cells that were lacking pJUN accumulation than ONC (Fig. 6H) (49.5% v 19%, P < 0.0001, χ^2^ test). Our interpretation of these data is that ONC can induce BAX translocation through both DLK-JNK-dependent and independent pathways, and that the independent pathway is enriched by direct inhibition of FAK.

We also noted that injection of PF573228 was associated with a propensity to affect larger cells and was statistically similar to the population of cells affected within 24 hrs of ONC (Fig. 6I). By 4–5 days pONC, GFP-BAX translocation was present in cells that were statistically smaller (P < 0.0003 relative to 1 day post-ONC and 1 day PF573228 treatment, ANOVA, data not shown). Interestingly, a small proportion of RGCs in both PF573228-treated eyes, and after 24 hrs post-ONC, exhibited BAX translocation that was restricted to regions of the dendritic arbor (Fig. 6J) including different secondary branches off the same primary branch.

**Figure 7:**
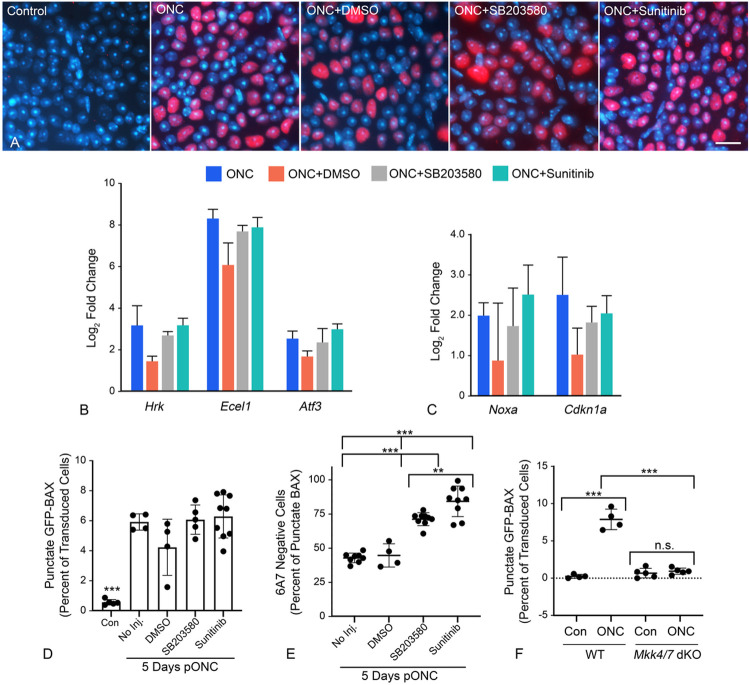
The effect of kinase inhibitors on the processes of BAX translocation and activation. Two inhibitors that are predicted to affect elements of the DLK signaling axis (SB2035580 to inhibit p38/MAPK14 and Sunitinib as a broad spectrum inhibitor) were injected into eyes immediately after optic nerve crush (ONC) surgery. (A) Immunostaining for nuclear accumulation of pJUN, 1 day after ONC surgery, shows both inhibitors have no effect on this surrogate for JUN activation. Scale bar = 15 μm. (B, C) Quantitative PCR analysis of pJUN (B) and p53 (C) target genes 5 days after ONC. Similar to pJUN staining results, neither of the inhibitors prevented normal increases in transcript abundance from both transcription factors. (D) Quantification of the percentage of transduced cells exhibiting GFP-BAX translocation at 5 days after ONC. Neither inhibitor suppressed the translocation response, which were significantly increased relative to contralateral eyes (Con) (***P<0.0001 for each ONC group in individual comparisons by *t*-test). (E) Analysis of BAX activation by 6A7-immunostaining showed that both inhibitors suppressed permeabilization of translocated BAX relative to ONC alone or ONC with DMSO vehicle injection (ANOVA, ***P<0.0001). Notably, Sunitinib significantly reduced BAX permeabilization relative to SB203580 (*t* test, **P=0.0053) even though it is not reported as a p38/MAPK14 inhibitor (73). (F) Quantification of the percentage of transduced cells exhibiting GFP-BAX translocation 5 days after ONC, comparing *Mkk4^fl^;Mkk7^fl^* Tg mice without prior introduction of Cre recombinase by AAV2-mediated gene transduction (considered wild type, WT) with *Mkk4^fl^;Mkk7^fl^* mice exposed to AAV2-Cre prior to crush surgery (*Mkk4/7* dKO). WT mice exhibit an ONC-induced increase in cells showing translocation relative to contralateral eyes (***P<0.0001). Mice conditionally lacking function MKK4 and MKK7 exhibit significantly fewer punctate cells after ONC, relative to WT animals (***P<0.0001) and no discernable change in translocating cells relative to contralateral eyes (n.s., not significant).

Since FAK inhibition-induced GFP-BAX translocation could be attributed to only a subset of RGCs, we explored further if the DLK-JNK axis affected either translocation and/or permeabilization after ONC. We tested whether two inhibitors could affect BAX activation if immediately injected after ONC surgery. First, SB203580, a selective p38/MAPK14 inhibitor which is also implicated in anoikis (72) and second, sunitinib, an inhibitor with broad selectivity which has been used to disrupt the DLK-JNK axis (36). Importantly, sunitinib does not affect p38/MAPK14 (73). Assessment of each inhibitor to suppress the DLK-JNK axis was made by staining for pJUN nuclear accumulation as a surrogate for activation of the axis. At 24 hrs after ONC neither inhibitor prevented the accumulation of pJUN (Fig. 7A), nor prevented the expression of either pJUN- or p53-mediated gene expression at 5 days post-ONC (Figs. 7B, C). While no effect on the DLK-JNK axis was expected for p38/MAPK14 inhibition, this result was unexpected for sunitinib. GFP-BAX translocation after 5 days was unimpeded by both inhibitors relative to ONC surgery alone (Fig. 7D), but both significantly attenuated BAX permeabilization as a function of 6A7-staining (P < 0.0001, ANOVA, Fig. 7E). Sunitinib yielded a greater effect at preventing permeabilization than the p38/MAPK14-selective inhibitor SB203580 (P = 0.0053, *t*-test).

Since sunitinib had no effect on blocking the DLK-JNK axis, we instead interrogated the relative contribution of this pathway in mice with conditional ablation of both *Mkk4* and *Mkk7* genes, which encode two kinases central to the axis (38). To avoid the developmental defects associated with combined *Mkk4/7* deletion (38), these genes were targeted in adult mice using AAV2-mediated delivery of Cre recombinase at the time cells were also transduced with AAV2/2-*Pgk*-GFP-BAX. At 5 days post-ONC, *Mkk4/7* deficient cells exhibited significantly fewer cells exhibiting GFP-BAX translocation compared to wild type mice (P < 0.0001, *t*-test) and no increase in punctate cells relative to contralateral uncrushed retinas (Fig. 7F). Together, these data indicate that the DLK-JNK axis is a critical component in facilitating the translocation of GFP-BAX in a majority of RGCs but is insufficient to stimulate permeabilization in the absence of active kinases such as p38/MAPK14.

## Discussion

To our knowledge this study represents the first kinetic analysis of BAX translocation and permeabilization in cells present in a complex tissue environment. The process of translocation can be initiated several days after the initial damage to the RGC axon, but once initiated proceeds rapidly in RGCs albeit with statistically slower kinetics than we have observed in tissue culture cells. Slower translocation of BAX to the MOM may be a consistent feature of RGCs regardless of the apoptotic stimulus, including exposure to STS, a kinase inhibitor that has frequently been used to assess the kinetics of BAX activation in tissue culture cells. This may be a phenomenon that is intrinsic to RGCs, but at this time we cannot rule out that this process is influenced by surrounding cells, such as supporting macro- and microglia that are responding to the damaged environment. Such responses could be the generation of trophic factors, clearance mechanisms of damaged RGC compartments, and the efforts to maintain ion homeostasis and synaptic function (74, 75, 76).

We observed translocation of GFP-BAX in several RGC compartments, including the dendritic arbor and both the intraretinal and optic nerve axon. This observation suggests that BAX-mediated catabolism may play a role in degeneration of these compartments and could explain why axonal degeneration in glaucomatous DBA/2J mice is delayed in the absence of a functional *Bax* gene (26). BAX and/or caspase-dependent axonal degeneration has been described in several neuronal cell types (77, 78, 79) and may precede SARM-1-mediated calpain-dependent catabolism, observed in other cell types (80). Similarly, the pruning of dendritic branches has been linked to caspase activation (81, 82) and recent studies suggest that arbor remodeling in some RGCs is mediated by a BAX-dependent mechanism (83).

We also observed a small percentage of cells with punctate GFP-BAX restricted to the dendritic arbor in both ONC retinas and in retinas treated with the FAK inhibitor PF573228. The arbor may be a critically susceptible region of the RGC where the process of BAX translocation is initiated and may account for why larger RGCs, which have the largest and most complex dendritic arbor branching (84), were the earliest to exhibit BAX translocation in both ONC and FAK inhibition experiments. We speculate that once initiated, BAX translocation may then propagate to other compartments of the RGC. The propagation of apoptosis, including BAX translocation, MOMP, mitochondrial membrane depolarization, and caspase activation has been described in tissue culture cells and some neurons (85, 86, 87, 88, 89, 90, 91, 92, 93). With respect to BAX translocation in tissue culture cells, propagation is initiated preferentially in distal tips of processes and then travels to the soma and even into additional processes at a velocity of ~ 7 μm/sec (85). Within the soma, however, BAX translocation initiates simultaneously at mitochondrial foci. If RGCs also propagate BAX translocation at a similar rate, this would explain why we observed so few cells with punctate BAX restricted to the arbor, suggesting we were capturing a rare early stage of propagation. The concept that BAX translocation is initiated first in the dendritic arbor aligns with observations made by others that remodeling of this compartment represents one of the earliest morphological changes associated with RGC pathology after optic nerve damage (94, 95, 96, 97).

Our results also showed that the processes of BAX translocation and permeabilization in RGCs were separated by a significant delay. This contrasts dramatically from studies in tissue culture cells undergoing BAX-dependent apoptosis, where BAX permeabilization leading to MOMP occurs virtually simultaneously with the translocation of the first BAX molecules (11, 98, 99). The precise length of the delay interval in RGCs was not clear from our experiments, but we posit that it may range from several hours to days. Longitudinal imaging of GFP-BAX translocation in eyes of mice subjected to ONC suggests that cells with translocated BAX can remain present for up to 5 days before being cleared (32).

A small proportion of RGCs exhibited first translocation of BAX followed immediately by its retrotranslocation back into the cytosol, a phenomenon that has also been documented in detached tissue culture cells that have initiated anoikis, but are then allowed to reattach with the surrounding matrix (14, 72). Studies on anoikis suggest that the process is initiated by the loss of function of FAK. In this pathway, FAK inhibition leads to the activation of the latent BH3-only protein BMF (66, 100). Anoikis also exhibits a delay between translocation and permeabilization that has been estimated to be up to 3–4 hrs. We hypothesized that a similar mechanism may be a critical feature of some damaged RGCs. Consistent with this, we observed a significant increase in BAX translocation in naïve retinas exposed to the FAK inhibitor PF573228. Importantly, PF573228 induced a similar level of translocation after 24 hrs as ONC at this time point but did not induce high levels of BAX permeabilization. We interpret the effect of PF573228 as priming BAX in RGCs without necessarily activating it. This would explain previous observations that FAK inhibition exacerbates RGC loss but only after optic nerve damage (101, 102).

The mechanism of BAX translocation is still not elucidated but is likely tied to the activity of BH3-only proteins. A predicted primary function of BH3-only proteins is that they sequester anti-apoptotic proteins like BCLX_L_ allowing for a shift in the cytosolic-MOM equilibrium of pro-apoptotic counterparts (103). This concept is reenforced by data showing the BH3-only proteins have a stronger binding affinity to anti-apoptotic members, and only exhibit transient interactions with pro-apoptotic proteins (103, 104). BH3-only proteins, however, are also thought to play a direct role in BAX translocation. Evidence suggests that some BH3-only members bind to the surface of inactive BAX dimers in the cytosol to facilitate their separation into monomers and translocation to the MOM (105, 106). This form of BAX exposes the C-terminal membrane anchor domain allowing it to associate with Voltage-dependent anion channel type 2 (VDAC2) on the MOM. This is also the likely state of BAX and BAK in healthy cells when they are in the BCLX_L_/BCL2-mediated equilibrium with the cytosol, and we predict, the state of translocated BAX in affected RGCs that have not undergone permeabilization.

Protein structural studies also indicate that BH3 mimetics play a role in the BAX permeabilization step. Mimetics can bind to a region of BAX distinct from its BH3-domain causing it to undergo a conformational change leading to unfolding of the “latch” domain that exposes hydrophobic alpha-helices that further strengthen the MOM-BAX interaction (4, 107) and the initiation of proteolipid pores. This model is difficult to reconcile with our results using kinase inhibitors. Both SB203580 and sunitinib failed to block the expression of BH3-only genes that are linked to the DLK-JNK axis (*Hrk* and *Noxa*) suggesting that these BH3-only proteins (and presumably *Bbc3* and *Bim*, which are also tied to p53 and pJUN activation, respectively) were active in these experiments, yet we still observed a significant attenuation of BAX permeabilization. Additionally, combined deletion of *Mkk4/7*, which inhibits the expression of these BH3-only genes, effectively blocked translocation of BAX in a significant proportion of cells. Therefore, our results are more consistent with BH3-only proteins playing a critical role in the translocation of BAX but are, by themselves, not sufficient for permeabilization.

The mechanism of retrotranslocation in damaged RGCs is also not clear. In healthy cells, BAX is retrotranslocated by BCLX_L_ (1), but it is unknown if this same mechanism is active in reattached cells during anoikis or in RGCs that exhibit spontaneous retrotranslocation. Overexpression of BCLX_L_ in both tissue culture cells and RGCs prevents BAX translocation in the first place (21), making study of its role in retrotranslocation difficult. We predict a model, however, in which a subset of RGCs undergoing anoikis are able to recover cell-cell contacts and have sufficiently high enough levels of endogenous BCLX_L_ to initiate retrotranslocation. Further studies to explore this model are ongoing.

The permeabilization of the MOM by translocated BAX in anoikis is linked to the activity of p38/MAPK14 (72), although it is not clear if this kinase modifies BAX directly, or if the mechanism is indirectly linked to p38/MAPK14 modification of an intermediate substrate. A recent report implicates DRP1 as directly interacting with BAX and mediating its activation (108) and p38/MAPK14-dependent phosphorylation of DRP1 has been linked to other neurodegenerative conditions (109, 110). p38/MAPK14 has previously been shown to have a role in RGC pathology after both acute and chronic optic nerve damage (111, 112), but the actual mechanism of neuroprotection afforded by blocking this kinase has not been studied in detail. Blocking p38/MAPK14 with SB203580 had no impact on preventing BAX translocation, but it did suppress the permeabilization of translocated BAX up to 5 days after ONC. The inability to block translocation contradicts previous reports that p38/MAPK14 is required for this process in cultured cells (113, 114, 115), although these studies relied on cell fractionation experiments or used antibodies that only recognized permeabilized BAX. Alternatively, the difference between these previous observations and ours could be associated with a difference in activation mechanisms between isolated cultured cells and RGCs in a complex tissue.

Interestingly, we also observed that a low dose of sunitinib also blocked BAX permeabilization, but not translocation. Sunitinib is a broad-spectrum kinase inhibitor, but does not directly affect p38/MAPK14 activity (73). Additionally, at the concentration we used, we did not affect activation of the DLK-JNK axis, thus modulation of either MKK4/7 or JNK2/3, both of which have been linked to the activation of p38/MAPK14 (116), would not be expected to be affected in our experiments. The implication of this is that low levels of sunitinib were affecting one or more kinases that have yet to be identified in the BAX activation process. Based on known selectivity measures (73), the concentration of sunitinib we used could target as many as 99 other known kinases before reaching efficacy to inhibit DLK. Identification of other kinases in the BAX activation process in RGCs is ongoing.

Acute damage to the axons of the optic nerve leads to nearly complete loss of the entire RGC population in the mouse retina (117), but elegant studies using single cell RNA-Seq clearly show that RGC populations exhibit varying levels of susceptibility to this damage paradigm (118). One interpretation of these differences in susceptibility is that damage induced by ONC is not monolithic and is instead multifactorial. For example, some RGCs may be susceptible to loss of FAK signaling, while others are more susceptible to ER stress or accumulation of reactive oxygen species, etc, as part of a continuum of damaging stimuli. Since *Bax* deletion blocks essentially all RGC soma death after optic nerve injury (23, 24, 26) it is likely the common denominator of all the proapoptotic signaling pathways in these cells after optic nerve damage. Consequently, we predict that an analysis of the pattern of BH3-only protein activation/expression would act as a signature of the critical damaging stimulus of a given RGC subtype, consistent with studies that show that the pattern of BH3-only proteins activated in apoptosis varies with the type of stimulus (5, 119). Ultimately, final susceptibility may be a function of the cooperation of different members of the BH3-only protein family. For example, RGCs susceptible to FAK inhibition may be more sensitive to anoikis-driven BMF activation, which would account for why a higher proportion of cells exposed to PF573228 exhibited punctate GFP-BAX without DLK-JNK driven accumulation of pJUN and its associated BH3-only proteins. Variable RGC susceptibility as a function of BH3-only protein levels also explains observations that selective deletion of these genes only provides partial protection to RGCs after ONC (41, 44, 45, 46, 47, 48). The implication of a model in which RGC death is a function of multiple different, and possibly sequential stimuli, is that there is no single early molecular event that can be targeted therapeutically, complicating the development of a global neuroprotective strategy. Conversely, targeting later BAX activation, particularly the permeabilization step, could offer promise as a strategy to facilitate recovery of RGCs after damage.

## Conclusions

Acute optic nerve damage induces BAX activation which can be temporally separated into a stage of BAX translocation to the MOM and a stage where it permeabilizes the MOM leading to the release of cytochrome c. Translocation is associated with the ability of the cells to express and/or activate BH3-only proteins, while permeabilization is associated with one or more kinases, including p38/MAPK14. At least of subset of RGCs exhibit sensitivity to loss of signaling pathways linked to cell-adhesion and FAK activity similar to the process of anoikis.

## Methods

### Animals

Adult BALB/cByJ or C57BL/6J mice (Jackson Laboratory, Bar Harbor, ME) were housed in microisolator cages with a 4% fat diet (8604 M/R; Harland Teklad, Madison, WI) and maintained in a 12 hour light/dark cycle. C57BL/6J mice carrying dual floxed alleles of *Mkk4* and *Mkk7* were developed as previously reported (38). Biological variability involving sex-related differences in response to optic nerve damage was considered in the design of experiments. While no sex differences have been detected using the acute ONC protocol (51), experimental groups were each assigned an equal mixture of male and female mice. Animals were handled in accordance with the Association for Research in Vision and Ophthalmology statement on the use of animals in research. The Institutional Animal Care and Use Committees of the University of Wisconsin and the University of Rochester approved the experimental protocols and ethical care for mice used in these experiments. Any animals that exhibited distress were removed from the study. Euthanasia was performed by intraperitoneal injection of 0.05 mL of Euthasol (Virbac AH, Fort Worth, TX). Following cessation of a detectable heartbeat, mice were cervically dislocated.

### Optic nerve crush

ONC was performed as previously described (52, 120). Briefly, mice were anaesthetized via intraperitoneal injection of ketamine (120 mg/kg) and xylazine (11.3 mg/kg). One drop of 0.5% proparacaine hydrochloride (Akorn, Lake Forest, IL) was used to numb the experimental eye. A lateral canthotomy exposed the optic nerve and was followed by an incision in the conjunctiva at the limbal junction. The optic nerve was clamped for 3 seconds using self-closing N7 forceps (Roboz Surgical Instrument Co, Gaithersburgs, MD). After surgery, triple antibiotic ointment was used to cover the eye, and a subcutaneous injection of buprenorphine (0.2 mg/kg) was delivered to mitigate pain. The right eye was not surgerized and evaluated as the contralateral condition.

### Intravitreal injections

After anesthesia as described above, a sterile 30G needle was used to puncture a small hole through the conjunctiva and scleral tissue. A beveled 35G Nanofil needle affixed to a Nanofil syringe (World Precision Instruments, Inc, Sarasota, FL) was inserted in the pre-poked hole to deliver desired virus, drug or vehicle. The contents were slowly delivered over 30 seconds, and the needle remained within the globe for an additional 30 seconds with caution, such that the lens was not damaged. Antibiotic ointment was placed over the injection site and buprenorphine (0.2 mg/kg) was administered to relieve pain. AAV2/2-*Pgk*-GFP-BAX, AAV2/2-*Pgk*-mCherry-BAX, and AAV2/2-*Pgk*-Cytochrome c-GFP were packaged by the University of North Carolina (UNC) Vector Core (Chapel Hill, NC) (1–5 x 10^12^ genome copies/ml). AAV2/2-CMV-Cre virus (> 10^12^ gc/mL) was purchased from the UNC Vector Core. All injections of virus were conducted at least 1 month prior to conducting optic nerve crush surgery.

The following inhibitors were diluted in sterile DMSO for intravitreal injection; FAK inhibitor PF573228 (Tocris Bioscience, Bristol, UK), p38/MAPK14 inhibitor SB203580 (Millipore Sigma, St. Louis, MO), and DLK inhibitor Sunitinib (Selleckchem, Houston, TX). Experimental eyes received 1 μL of PF573228 (10 μM – 25 mM), 25 μM SB203580, or 10 μM Sunitinib, while the contralateral eyes received 1 μL DMSO-only injections. If mice received an inhibitor injection the same day they underwent ONC, the intravitreal injection was performed immediately following the ONC procedure. For STS treatment, eyes were injected with injected with 0.6 μL of a 10 μM solution to yield an approximate final concentration of 1 μM STS.

### Clones and plasmids

The GFP-BAX construct used for tissue culture cell experiments was previously described (121). The HDAC3-Flag construct was a gift from Eric Verdin (Addgene plasmid no. 13819: Addgene, Cambridge, MA). The mitoBFP construct was described previously (11, 53). Transgenes in these plasmids were all driven by the CMV promoter. Plasmids used for generating AAV2 viral particles were derived from the parent plasmid AAV-*Pgk*-Cre (Addgene plasmid no. 24593), a gift from Dr. Patrick Aebischer. The coding sequence for Cre was removed by digestion with EcoRI and SalI and was replaced with a sequence bridge of single cutting restriction sites. This bridge vector was used to subclone GFP-BAX (121), mCherry-BAX (which was generated in the laboratory by subcloning BAX into pmCherry-C1, Clontech/Takara Bio, Kusatsu, Shiga, Japan), and cytochrome c-GFP (Addgene plasmid no. 41182), a gift from Douglas Green. All plasmids were validated by sequencing.

### Tissue culture cell maintenance

661W cells (mouse retinal progenitor cells) were cultured in DMEM (Cellgro, Mediatech, Inc., Manassas, VA) supplemented with 10% FBS (Atlanta Biologicals, Norcross, GA) and 1% Antibiotic/Antimycotic (Cellgro, Mediatech, Inc.) at 37°C and 5% CO2. Treatment with 316nM staurosporine for 24 hours induced 661W differentiation (122). For transfection, prewarmed media containing 2 μg each of the GFP-BAX, mitoBFP, and HDAC3-Flag plasmids was mixed with 2 volumes of Transfast reagent (E2431, Promega, Madison, WI), vortexed and incubated at room temperature for 15 min. After removal of regular media, the transfection mixture was added to cells and they were incubated at 37°C for 1 hr, after which fresh media was added to the cells and they were incubated for a further 15-24hr. Overexpression of HDAC3-Flag is selectively toxic to differentiated neurons (123), including differentiated 661W cells (31) and plays a critical role in the apoptotic program executed by RGCs (56, 58). 661W cells, originally described as RGC-5 cells from *Rattus* norvegicus, were confirmed in our laboratory as *Mus musculus* origin by PCR of the mitochondria d-loop (124) and the upregulation of TUBB3 after differentiation (31).

### Live imaging and analysis

Live-cell imaging was performed using an Andor Revolution XD spinning disc confocal microscopy system (Andor, Belfast, Northern Ireland) comprised of the Nikon Eclipse Ti inverted microscope, Nikon objectives, an iXon x3 897 EM-CCD camera, a Yokogawa CSU-X1 confocal spinning disk head, the Andor Laser Combiner with four solid state lasers, an ASI motorized stage with Piezo-Z, and an Okolab CO_2_ cage incubator for temperature and CO_2_ control at 37°C and 5% CO_2_.

661W cells were plated at a density of 100,000–300,000 cells per well on 4-well chamber slides containing #1.5 optical grade plastic (Ibidi, Madison, WI). Cell treatments were staggered for an appropriate time for the cell type to ensure the ability to image each well on a slide. Prior to imaging, phenol-containing media was removed from the well, and replaced with a recording media prepared in-house (HBSS with 1.26 mM calcium chloride, 0.49 mM magnesium chloride, 0.4 mM magnesium sulfate, no phenol red, 4.5 g/L glucose and 10 mM HEPES) supplemented with appropriate serum concentration. Under temperature and CO_2_ control, time-lapse imaging was performed by collecting z-stacks consisting of 20–25 optical sections at 0.22μm (Nyquist) taken every minute for one to two hours.

Live *ex vivo* imaging of mouse retina was performed with the same microscopy conditions as discussed above. To prepare the retina, animals were euthanized with a lethal overdose of pentobarbital sodium, stained with live-cell nuclear dye DRAQ5 (ThermoFisher Scientific, Waltham, MA), then mounted on a coverslip bottom (#1.5) chamber slide (Ibidi). For DRAQ5 staining, once euthanasia was confirmed, an intravitreal injection of DRAQ5 was administered (200 μm final concentration), then the enucleated eye globe was incubated in nutrient rich medium at 37°C for 10 minutes. DRAQ5 labeling was stable for ~ 3 hours. To mount the retina, an eye cup was created from the eye globe, then a single relaxing cut through the retina and sclera was made in the anterior region. Without removing the dissecting scissors from the relaxed cut, in one quick motion, the lens was removed with the dissecting scissors as the retina was flipped such that the ganglion cell layer was facing down onto the coverslip portion of the chamber slide. A second relaxing cut was made, allowing the retina to rest as flat as possible. A drop of 2% low melt agarose (ThermoFisher Scientific) medium was placed on the retina, followed by a small piece of coverglass to aid in flattening the retina for imaging purposes. Once positioned, the retina was overlayed in the agarose medium, allowed to polymerize briefly, and covered with a layer of sterile water to prevent evaporation and image drifting during acquisition. The low melt agar was made in 50% ‘recording media’ (above) and 50% Neurobasal medium (ThermoFisher Scientific) supplemented with 1X Neurocult SM1 (Stemcell Technologies, Cambridge, MA) and 10% FBS (Atlanta Biologicals). The FBS and Neurocult supplements were added after the melted agarose had been cooled to 60°C. Both experimental and control retinas were mounted in the same way, and this process was completed in approximately 45 minutes from the time of euthanasia. Z-stacked images were collected every 5 minutes for up to three hours after the start of time-lapse imaging. DRAQ5 staining provided a control for tissue drift during imaging. Only RGCs that maintained a fixed position, based on nuclear visibility, were used for subsequent kinetic analyses.

For STS treatment, naïve animals were euthanized and the eyes injected with DRAQ5 and STS to a final concentration of 1 μM and incubated at 37°C for 10 min. The retinas were then harvested and mounted for imaging as described above except that the media contained 1 μM STS during the imaging procedure.

All images were subject to the same background subtraction and Gaussian filtering within the IMARIS 7.7 software (Bitplane, Concord, MA), where each filter had its own constant algorithm and threshold setting. The spots function was used to identify and track GFP-BAX foci within a cell and extract the maximum fluorescence of the desired fluorophore from each surface. A subset of BAX foci was selected for computational analysis based on the longevity of the track, whether the track encompassed the translocation event and remained within the defined 3-dimensional space of the cell, and whether the BAX foci were spatially isolated. For ONC experiments, a total of 33 cells, from 7 retinas, exhibiting translocation during an imaging session were recorded. Of these cells, we were able to extract kinetic data for a minimum of 3 mitochondrial foci per cell for 27 cells (5 of which also exhibited retrotranslocation). For STS treated retinas, we were able to record 10 cells from 4 retinas, all of which were suitable for kinetic analysis.

### Computational Analysis

BAX translocation analysis was performed as described previously (11). Briefly, raw maximum fluorescence data extracted from the IMARIS program was normalized (to its initial value) for each foci within a cell to allow for comparison among many GFP-BAX foci. These data were then fit to a sigmoid curve using a linear regression model. The code using the SciPy library in the Python programming language is available in (11). Quantification for retrotranslocating BAX cells was performed by applying sigmoid functions to each phase separately (translocation and retro-translocation). The retrotranslocation curve was first fit using a gaussian function to determine the maximum point of the curve, which would define the two phases.

### Retinal tissue preparation and immunostaining

Enucleated eyes were punctured on the anterior portion of the limbus with a 30 gauge needle and then immersed in 4% paraformaldehyde in PBS for 1 hour. Eye cups were made by removing the anterior chamber of the eye by cutting along the limbus. In preparation for immunofluorescent staining, retinal eye cups were first wiped with the tip of a twisted Kimwipe to remove the residual vitreous and then equilibrated in 30% sucrose in PBS overnight at 4°C. They were then subjected to 3 rounds of freeze-thawing on dry ice and blocked in PBS containing 2% goat serum and 0.1% Triton X-100 overnight at 4°C. Primary antibodies were added to the same buffer and incubated with the eyecups for a minimum of 3 days at 4°C. After incubation, the eyecups were washed 3 times in PBS and then incubated in PBS containing 2% goat serum and 0.1% Triton X-100 overnight at 4°C.

For immunostaining longitudinal sections of the optic nerve, fixed eyecups containing ~ 1 mm of the optic nerve were equilibrated in 30% sucrose in phosphate buffered saline (PBS) (50 mM phosphate, pH 7.4, 150 mM NaCl) overnight at 4°C and then embedded in Optimal Cutting Temperature media (Scigen Scientific, Gardena, CA) on dry ice. Cryosections (10 μm) were selected that included the optic nerve head and an extended portion of the optic nerve. These were first incubated in PBS at room temperature for 10 min and then blocked in PBS containing 10% horse serum (Lonza, Basel, Switzerland) and 0.1% Triton X-100 for 1 hr at room temperature in humidified chambers. Sections were then covered in PBS containing 2% BSA and 0.1% Triton X-100 with primary antibody (SMI-31 see below) overnight at 4°C. After incubation, the sections were washed 3 times in PBS and incubated in the same buffer (PBS, 2% BSA, 0.1% Triton X-100) with the secondary antibody for 1 hr at room temperature.

The following primary antibody and corresponding concentrations were used: chicken anti-GFP polyclonal (1:1000) (no. ab13970, Abcam, Cambridge, UK), rabbit anti-mCherry polyclonal (1:500) (no. ab167453, Abcam), mouse anti-BAX (1:50) (6A7) monoclonal (no. sc-23959, Santa Cruz Biotechnology, Dallas, TX), rabbit anti-pJUN (Ser73) monoclonal (1:500) (D47G9) (no. 3270, Cell Signaling Technology, Danvers, MA), and mouse anti-phospho-neurofilament monoclonal (1:1000) (SMI-31) (no. NE1022, Millipore Sigma). Eyecups were then washed in PBS and incubated in a solution containing secondary antibody overnight at 4°C. The following secondary antibodies were used at a concentration of 1:1000: polyclonal goat anti-mouse antibody that was conjugated to AlexaFluor-594 (Jackson ImmunoResearch Laboratories, West Grove, PA), polyclonal donkey anti-chicken conjugated to AlexaFluor-488 (Jackson ImmunoResearch), polyclonal goat anti-rabbit antibody conjugated to AlexaFluor-594 (Jackson ImmunoResearch). Following incubations with secondary antibodies, both eyecups and sections were washed 3 times in PBS. For whole mounting, the retinas were isolated from the eyecups and placed on glass Superfrost Plus slides (ThermoFisher Scientific) with 4 relaxing cuts to help flatten the tissue. Both sections and retinas were then mounted using Vectashield containing DAPI (Vector Laboratories, Burlingame, CA), coverslipped, and sealed with clear nail polish. Slides were protected from light and stored at 4°C until image acquisition.

### Fixed tissue imaging and analysis

For all images used for quantification, samples were imaged on a Zeiss Axioimager Z2 upright microscope (Zeiss, Oberkochen, Germany). All images were analyzed using Zen Blue software (Zeiss). Separate imaging modalities were used to quantify the percentage of cells with punctate GFP-BAX and the percentage of 6A7 positive cells. To quantify the percentage of GFP-BAX labeled cells with punctate GFP-BAX, 4 images per retinal quadrant were taken using a 20X air objective. The retinal eccentricity was held constant in the mid-periphery. For those 16 images, every GFP-BAX positive cell was counted. A minimum of 700 cells were counted for each retina. To quantify the percentage of 6A7 positive cells, a second set of images was taken of each sample. Beginning at one side of the retina, the camera was panned across the retina, and every field that contained a cell with punctate GFP-BAX was imaged. This was necessary to maximize the number of cells with punctate GFP-BAX counted per retina. An average of 94 punctate cells were counted for each retina with a range of 33–177 cells counted. The images of GFP-BAX puncta in the dendritic arbors of RGCs were imaged on a Leica SP8 (Leica Camera, Wetzlar, Germany) confocal microscope using a 1.4 NA 63X oil objective.

### Realtime quantitative polymerase chain reaction

Immediately after euthanasia, eyes were enucleated and the retinas were extracted and flash frozen in microcentrifuge tubes on dry ice. A total of 12 retinas were used for each experimental group. These were split into groups of 3 and their OS and OD retinas were frozen in separate tubes. Therefore, each experimental group in the experiment consists of four replicate values obtained from three mice each. RNA was extracted from the pooled retinas using a DNA/RNA/Protein extraction kit (IBI Scientific, Dubuque, IA). RNA quality was verified using a Nanodrop One spectrophotometer (ThermoFisher Scientific). All RNA samples had a A260/280 value between 2.10 and 2.13 and an A260/230 value between 2.00 and 2.30. cDNA was created by annealing 2 μg of total RNA from each sample to Oligo(dT)_15_ Primers (Promega) and then incubating with MMLV-RTase (Promega) in a 40°C water bath for 1 hr. The 25 μL total volume of each reaction was diluted by addition of 200 μL of diethyl pyrocarbonate treated, nuclease free water (ThermoFisher Scientific). Quantitative PCR was performed in a Quant Studio 7 Flex Real Time PCR machine (ThermoFisher Scientific) using a customized TaqMan Array Card (ThermoFisher Scientific) that was designed to assess the abundance of the following mouse RNA transcripts: *18S, Hrk, Ecel1, Atf3, Noxa*, and *Cdkn1a (p21)*. Changes in transcript abundance were calculated using the ΔΔC_t_ method (125) using the 18S rRNA value from each sample as a reference to calculate ΔC_t_ and then the change caused by the experimental paradigm was calculated as the difference in ΔC_t_ values between the experimental eyes and the contralateral eyes.

### Statistical analysis

Calculations of sample size for statistical power were made using previous data on outcome metric variances for ONC experiments. Cohort sizes of n = 5 retinas provide a power set to 0.8 to detect a difference of 12% between groups and n = 4 retinas to detect a 20% difference. Translocation rates from live-cell imaging experiments did not fit a normal distribution and were Log_2_ transformed for analysis. One-way analysis of variance (ANOVA) was used to evaluate differences when comparing multiple groups. A two-sided Student’s *t*-test was used to compare the means of two isolated cohorts. A χ^2^ test was used to compare the proportions of punctate GFP-BAX cells showing accumulation of pJUN after ONC or injection of PF537228. To assess the intracellular synchronicity of both the time of initiation and the rate of translocation, we used data collected from multiple points within a single cell to determine the coefficient of variation (CoV) for each variable per cell. These statistics were normalized to a percentage (absolute value) of the mean for each cell within a single group to facilitate comparison between groups. Data was generally represented as scatter plots showing mean ± standard deviation in graphs. Each data point represents the value obtained from an individual retina unless otherwise specified. For clarity, larger datasets were graphed as Box and Whisker plots showing the mean as a red line ± standard deviation. Individual data points outside of the standard deviation were included as individual points on each graph.

## Figures and Tables

**Table 1:** Comparison of BAX translocation kinetics between tissue culture cells and retinal ganglion cells (RGCs) in *ex vivo* retinal imaging experiments. Apoptotic stimuli included staurosporine (STS), overexpression of histone deacetylase 3 (HDAC3), or optic nerve crush (ONC). “Zero times” for the initiation of translocation are based on either the addition of STS (D407, HeLa, HCT116 cells, and the RGC STS cohort), time from transfection of cells (661W cells), or time of animal euthanasia (RGC ONC and Retro cells). Retinal explants were harvested from eyes at 7 days after ONC (naïve retinas were used for STS experiments). The initiation times and translocation rates were calculated from the translocation data after fitting with a sigmoid function as previously described [11]. Means and standard deviations are derived from cell populations, where multiple foci within a cell were first averaged, and cells with fewer than 3 foci were omitted. Values for Coefficient of Variation within the cell population were calculated as described in the Methods. Y-Maximum refers to the number of relative fluorescence units (RFU) of fluorescently tagged-BAX that had accumulated at individual foci during an experiment. Values of RFU intensity were set to 1.0 at baseline. All data represents experiments using GFP-BAX.

Cell Type	Number of Cells	Number of BAX Foci	Apoptotic Stimulus	Initiation of Translocation (Minutes)	Coefficient of Variation (Initiation)	Translocation Rate (Log_2_RFU/min)	Coefficient of Variation (Rate)	Y- Maximum (RFU)
D407^a^	42	682	STS	174 ± 32	2.5%	−1.8 ± 0.8	55%	3.8
HeLa^a^	16	145	STS	158 ± 24	2.9%	−1.0 ± 0.6	154%	3.4
HCT116^a^	36	339	STS	551 ± 52	1.0%	−1.8 ± 0.6	55%	4.1
661W	20	342	HDAC3 Overexpression	795 ± 63	0.6%	−2.2 ± 0.9	46%	3.2
RGC ONC	22	160	ONC	99 ± 24	12%	−5.1 ± 0.9	22%	1.5
RGC STS	10	52	STS	113 ± 34	14%	−6.0 ± 0.9	13%	1.4
RGC Retro – ON	5	22	ONC	77 ± 24	9%	−4.7 ± 0.8	30%	1.3
RGC Retro – OFF	5	16	ONC	n/a^b^	n/c^c^	+ 9.0 ± 4.4	n/c^c^	−1.3

a Data for D407, HeLa, and HCT116 cells were described previously in reference [11].

b Not applicable (n/a). Retrotranslocation was found to occur immediately after completion of maximum translocation in these cells.

c Not calculated due to the low number of data points available.

## Abbreviations

AAV2/2: Adeno-associated virus serotype 2/genotype 2
ANOVA: Analysis of variance
BH3: BCL2 Homology Domain 3
Con: Contralateral
CoV: Coefficient of variation
DLK: Dual leucine zipper kinase
DMSO: Dimethylsulfate
FAK: Focal adhesion kinase
HDAC3: Histone deacetylase 3
JNK: Jun-N-terminal kinase
MKK: Mitogen activated protein kinase kinase
MOM: Mitochondrial outer membrane
MOMP: MOM Permeabilization
ONC: Optic nerve crush
p38/MAPK14: p38a/Mitogen activated protein kinase 14
pJUN: Phosphorylated JUN
pNF: Phosphorylated neurofilament
pONC: Post optic nerve crush
RFU: Relative fluorescence units
RGC: Retinal ganglion cell
STS: Staurosporine
UNC: University of North Carolina
